# The minimum information required for a glycomics experiment (MIRAGE) project: improving the standards for reporting glycan microarray-based data

**DOI:** 10.1093/glycob/cww118

**Published:** 2016-12-19

**Authors:** Yan Liu, Ryan McBride, Mark Stoll, Angelina S Palma, Lisete Silva, Sanjay Agravat, Kiyoko F Aoki-Kinoshita, Matthew P Campbell, Catherine E Costello, Anne Dell, Stuart M Haslam, Niclas G Karlsson, Kay-Hooi Khoo, Daniel Kolarich, Milos V Novotny, Nicolle H Packer, Rene Ranzinger, Erdmann Rapp, Pauline M Rudd, Weston B Struwe, Michael Tiemeyer, Lance Wells, William S York, Joseph Zaia, Carsten Kettner, James C Paulson, Ten Feizi, David F Smith

**Affiliations:** 2 Department of Medicine, Glycosciences Laboratory, Imperial College London, Du Cane Road, London W12 0NN, UK; 3 Department of Cell and Molecular Biology, The Scripps Research Institute, 10550 N Torrey Pines Road, La Jolla, CA 92037, USA; 4 Department of Chemistry, UCIBIO@REQUIMTE, Faculty of Science and Technology, NOVA University of Lisbon, Caparica 2829-516, Portugal; 5 Department of Surgery, Beth Israel Deaconess Medical Center, Harvard Medical School, Boston, MA 02115, USA; 6 Department of Science and Engineering for Sustainable Innovation, Faculty of Science and Engineering, Soka University, 1-236 Tangimachi, Hachioji, Tokyo 192-8577, Japan; 7 Biomolecular Frontiers Research Centre, Macquarie University, Sydney, NSW 2109, Australia; 8 Department of Biochemistry, Center for Biomedical Mass Spectrometry, Boston University, School of Medicine, 670 Albany Street, Suite 504, Boston, MA 02118, USA; 9 Department of Life Sciences, Faculty of Natural Sciences, Imperial College London, London SW7 2AZ, UK; 10 Department of Medical Biochemistry and Cell Biology, Institute of Biomedicine, Sahlgrenska Academy, University of Gothenburg, PO Box 440, 405 30 Gothenburg, Sweden; 11 Institute of Biological Chemistry, Academia Sinica, 128, Academia Road Sec. 2, Nankang, Taipei 115, Taiwan; 12 Department of Biomolecular Systems, Max Planck Institute of Colloids and Interfaces, Potsdam 14424, Germany; 13 Department of Chemistry, Indiana University, 800 E. Kirkwood Avenue, Bloomington, IN 47405, USA; 14 Complex Carbohydrate Research Center, University of Georgia, 315 Riverbend Road, Athens, GA 30602, USA; 15 Max Planck Institute for Dynamics of Complex Technical Systems, Bioprocess Engineering, 39106 Magdeburg, Germany; 16 NIBRT GlycoScience Group, NIBRT—National Institute for Bioprocessing Research and Training, Fosters Avenue, Mount Merrion, Blackrock, Co., Dublin, Ireland; 17 Department of Biochemistry, Glycobiology Institute, University of Oxford, Oxford OX1 3QU, UK; 18 Beilstein-Institut, Trakehner Str. 7-9, 60487 Frankfurt am Main, Germany; 19 Emory Comprehensive Glycomics Core, Emory University School of Medicine, Atlanta, GA 30322, USA

**Keywords:** glycan microarrays, glycans, glycobiology, glycomics, MIRAGE

## Abstract

MIRAGE (Minimum Information Required for A Glycomics Experiment) is an initiative that was created by experts in the fields of glycobiology, glycoanalytics and glycoinformatics to produce guidelines for reporting results from the diverse types of experiments and analyses used in structural and functional studies of glycans in the scientific literature. As a sequel to the guidelines for sample preparation (Struwe et al. 2016, Glycobiology, 26:907–910) and mass spectrometry  data (Kolarich et al. 2013, Mol. Cell Proteomics, 12:991–995), here we present the first version of guidelines intended to improve the standards for reporting data from glycan microarray analyses. For each of eight areas in the workflow of a glycan microarray experiment, we provide guidelines for the minimal information that should be provided in reporting results. We hope that the MIRAGE glycan microarray guidelines proposed here will gain broad acceptance by the community, and will facilitate interpretation and reproducibility of the glycan microarray results with implications in comparison of data from different laboratories and eventual deposition of glycan microarray data in international databases.

## Introduction

MIRAGE (Minimum Information Required for A Glycomics Experiment) is an initiative that was created by experts in the fields of glycobiology, glycoanalytics and glycoinformatics to produce guidelines for reporting results and facilitating the interpretation, evaluation and reproduction of data obtained from the diverse types of analyses used in structural and functional studies of glycans (http://www.beilstein-mirage.org). The history of this initiative and its three-component organization: coordinating group, working group and advisory board, have been reported previously (York et al. 2014).

Assignments of glycan structures as ligands or antigens increasingly depend on glycan microarray-based binding analyses, and accurate interpretation of results requires knowing the structures of the arrayed glycans. The preparation and characterization of the glycans depend on numerous techniques among them gel filtration, liquid chromatography, capillary electrophoresis, nuclear magnetic resonance and various types of mass spectrometry (MS). The information derived from the techniques used needs to be reported to enable a meaningful evaluation of the structure assignments. A working group comprises investigators, who have participated in the development and application of these methods, has been developing guidelines that are overseen by an advisory group and critiqued by the greater scientific community. These activities have already resulted in MIRAGE guidelines intended to improve the standards for reporting MS-based glycoanalytical data (Kolarich et al. 2013) and glycan sample preparation (Struwe et al. 2016).

There are similarities among DNA, protein and glycan microarray technologies, although the methods of analysis, the information sought and the conclusions from the different types of arrays are very different. Microarrays, being comprised of libraries of numerous elements (probes) that are simultaneously analyzed using many samples (binders), create unique challenges in documentation of data. Thus, early in the development of DNA arrays, Brazma and coworkers saw the need for a public repository for the data (Brazma et al. 2000). They realized that support of these databases would require major efforts in bioinformatics to capture the essential information, with definition of ontologies and formats to store the information, and tools for searching the databases. These considerations led to the development of “the Minimum Information for A Microarray Experiment (MIAME) that described the minimum information required to ensure that microarray data can be easily interpreted and that results derived from its analysis can be independently verified” (Brazma et al. 2001). This effort was successful and is predictably being applied to other technologies. Today, most data repositories for DNA expression based on arrays are compliant with MIAME, and the MIAME guidelines are now required to be followed for publishing in most scientific journals (Brazma 2009).

Investigations of protein–glycan interactions by studying glycan-binding proteins (GBPs), such as lectins and antibodies, and their binding to immobilized glycoconjugates or glycans have been conducted for decades (Magnani et al. 1980, Tang et al. 1985); however, the development of this approach as a high throughput method has required expansion of the library of glycans used for printing arrays. A pioneering effort in the area was the development of the procedure to convert reducing glycans to neoglycolipids (Stoll et al. 1990) that could be applied as 2 mm bands or 300 µm spots on silica gel TLC plates, nitrocellulose or PVDF membranes for monovalent immobilization and subsequently probed with biologically relevant GBPs (Fukui et al. 2002). A number of laboratories were active in developing the miniaturization of glycan arrays, which has also driven the development of synthetic and chemo-enzymatic approaches to expand libraries of glycans to populate large arrays (Magnani et al. 1980; Tang et al. 1985; Drickamer and Taylor 2002; Love and Seeberger 2002; Feizi et al. 2003; Schwarz et al. 2003; Ratner et al. 2004). However, it was the development of a microarray of 200 defined glycans (Blixt et al. 2004) and its evolution to over 600 glycans by the Consortium for Functional Glycomics (CFG) that generated much interest in this approach, in part due to the free services of the Protein–Glycan Interaction Service of the CFG that were made available to the scientific community through the NIGMS of the NIH (http://www.functionalglycomics.org).

Data from microarrays of defined glycans are generally used to determine the binding specificity of a given GBP by comparing the structural details of bound and non-bound glycans in the array. Such data have been extremely valuable in providing information on the specificities of GBPs that mediate host–pathogen interactions, innate and adaptive immunity and many other functions involving glycan recognition. The websites of CFG (http://www.functionalglycomics.org) and of the Glycosciences Laboratory at Imperial College London (https://www.imperial.ac.uk/glycosciences) contain information on the glycans available on their microarray platforms and summaries of the microarray binding data. Interpretation of the microarray data is dependent on the composition of the library of glycans printed on the array. Assignment of the ligand can best be made when the library contains a series of closely related glycan structures that are bound or not bound, but the conclusions are not necessarily unequivocal if the array does not contain the relevant glycome.

Ideally, the biological relevance of an assignment made by glycan array analysis should be evaluated by cellular or other in vivo analyses using the glycans assigned as ligands. Ultimately, the full spectrum of the biologically relevant determinants for a GBP can only be assessed with a glycan microarray presenting all possible natural glycans in the glycome in question. However, the largest glycan arrays of the CFG and Glycosciences Laboratory at Imperial College London have only 600–800 glycans, whereas the human glycome has been estimated to be comprising over 9000 glycan determinants (Cummings 2009). Thus, there is ample opportunity to expand glycan microarrays to more fully cover the diversity of structures in the human glycome. Glycan arrays containing glycomes are only recently becoming available and they enable the “preferred” natural ligands residing therein to be detected (Gao et al. 2014; Yu et al. 2014).

Apart from general screening analyses for defining the glycan determinants recognized by GBPs, glycan arrays are used as collections of defined glycan substrates for experiments to determine specificities of glycosidases and glycosyltransferases (Blixt et al. 2008; Chaubard et al. 2012); this involves the detection of specific alterations of the substrates following incubation with the enzymes. The use of glycan arrays for profiling anti-glycan antibody populations in serum is also of interest, as this could potentially lead to discoveries of glycan antigen determinants that are relevant to vaccine design, diagnostic assays and antibody-based therapies (Schneider et al. 2015; Muthana and Gildersleeve 2016).

As glycans become more readily available, interest in developing glycan microarrays has increased, and there have been several hundred articles published on this topic. However, there is no common experimental protocol, and many parameters involved in the design and production of glycan microarrays are unfamiliar to reviewers and editors of manuscripts reporting data using this technique. There are numerous methods of creating glycan arrays using various chemistries for attachment, different linkage structures (tags) and even post-immobilization modifications.

Here, we report guidelines (Supplementary data) intended to improve the standards for reporting data from experiments and analyses using glycan microarrays. These guidelines are intentionally minimal and apply only to information on generating glycan arrays and producing interpretable data for follow-on experiments. The purpose of these guidelines is not to cover every possible technique that can be used to create glycan microarrays, but rather to identify and highlight what parameters are important and should be reported in producing and analyzing glycan microarrays so that published data can be reliably interpreted by both the trained and untrained reader. We hope that the MIRAGE guidelines proposed here will gain broad acceptance by the community and thus will be as successful as the MIAME guidelines.

## Eight parts of MIRAGE glycan microarray guidelines

**Fig. 1.**
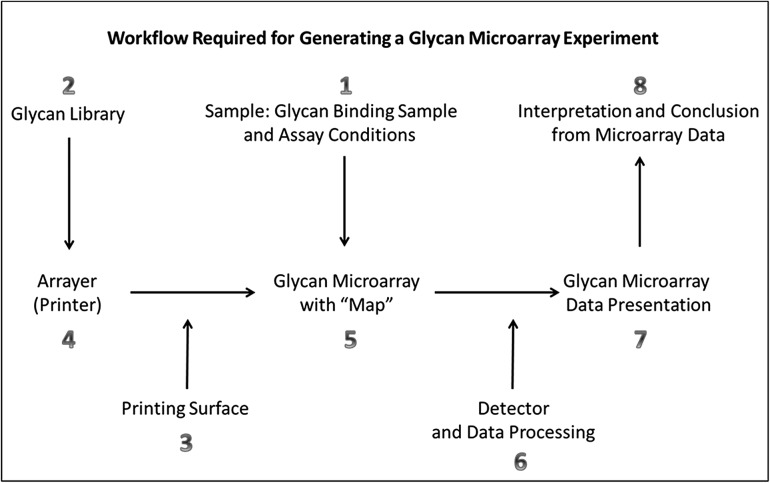
The eight major parts of the MIRAGE Glycan Microarray Guidelines (Supplementary data). Parts 1–6 mainly deal with generation of glycan microarrays, microarray binding experiments, detection and data quantitation, and Parts 7 and 8 focus on data presentation and interpretation in publications.

In developing MIRAGE guidelines for glycan microarrays, we attempted to follow the basic principles used for MIAME (Brazma et al. 2001), which required that the information about each experiment be sufficient to reproduce it, to interpret and compare results of similar experiments and be sufficiently structured so that data can be usefully queried, analyzed and mined. We designate eight components based on the workflow of a glycan microarray experiment (Figure 1).

The guidelines are provided in the Supplementary data and also on the website of the Beilstein-Institut (doi:10.3762/mirage.3). In brief, **Part 1** is about the glycan-binding sample. The term “Sample” is used for the entities being analyzed for glycan recognition throughout the guidelines. A wide variety of Samples can be applied onto glycan microarrays. The minimum information required includes description of Sample, modifications of Sample (if labeled for example) and assay protocols. **Part 2** is about the glycan library from which the glycan array is generated. The arrays may comprise glycans or glycoconjugates that are structurally defined; alternatively they may be partially purified and their structures unknown as in “shotgun” glycan arrays (Song et al. 2011; Yu et al. 2012, 2014; Byrd-Leotis et al. 2014); or they may be glycans in fractions on their way to being isolated from ligand positive macromolecules for characterization as in “designer” arrays (Palma et al. 2006, 2015; Gao et al. 2014). The guidelines under these parts include descriptions for defined and undefined glycans that are being interrogated in the arrays, as well as methods of modifications (functionalization or derivatization) of glycans before arraying process.

The properties of the surface used to present the printed glycans are covered in **Part 3**, and include types of surfaces, manufacturer information and custom preparation of surface where applicable. The method for immobilization (non-covalent or covalent) should also be described here. **Part 4** addresses the printing robot (arrayer or printer) used to deliver the glycans onto the array surface. Information should be provided on the instrument, dispensing mechanism, glycan deposition (volume and number of replicates of each glycan) and printing conditions including post printing treatment. **Part 5** is about the layout of glycans in the array. The minimum information required includes array geometry (e.g. single large array, subarrays, microtiter plate), numbers of spots for each glycan and in each array, identities of the printed glycans and methods for validating the identities (e.g. binding data from the array using Samples with known specificities). **Part 6** speaks to the means of detecting the binding and processing of the microarray data. Fluorescence scanning is currently the most commonly used detection method. The present version of the guidelines includes descriptions of scanning hardware, scanner settings (resolution, laser channel, photomultiplier (PMT) gain and scan power) and image analysis software used to quantify the output scanner image. The method used for data processing to obtain data in a table of results should also be described. **Part 7** and **Part 8** are about presentation of glycan array data and a brief comment on interpretation of data, respectively.

The majority of members of the MIRAGE Commission support the view that images of microarray experiments are not essential as minimal information at this time, but that the TIFF files, which represent the raw data, and accompany “detailed glycan map” [e.g. GenePix Array List (GAL) file, the text file with specific information about the location, size and name of each glycan spot on the array slide] and quantitation output files [e.g. proscan or GenePix Results (GPR) files], should be saved for future use once glycan array databases are available. The microarray images can be extremely informative with regard to assessing the background staining that can sometimes obscure positive results or even generate false positive results. Therefore, representative array images (reduced in size to accommodate easy transfer of data) can certainly strengthen the data in a manuscript or a database.

## Discussion

The MIRAGE guidelines aim to establish uniformity in the description of glycan microarrays and in the data collected without imposing rules on how the experiments should be performed. Applying the guidelines will not only facilitate interpretation and reproducibility of the results but also facilitate comparison of results obtained by different laboratories and eventual deposition of these results in databases. These will in turn enable development and use of data mining tools. Although databases presenting glycan array data are currently available online from the CFG (http://www.functionalglycomics.org) and the Glycosciences Laboratory at Imperial College London (https://www.imperial.ac.uk/glycosciences), they are not open to deposition of data from other glycan microarrays, nor are they readily comparable. These guidelines will stimulate the development of more universal tools as seen with the MIAME guidelines for RNA/DNA microarrays. For example, data submission tools will need to be developed enabling users to enter MIRAGE information directly into a repository or to export data in a standard format. A file format (digital standard format) with well-defined terms (standard representations: ontologies and dictionaries) for representing MIRAGE information in the computer will also be developed and this is among the next steps of the MIRAGE group. Efforts have been made to develop data mining software to discover glycan-binding motifs based on currently available glycan microarray data (Cholleti et al. 2012; Xuan et al. 2012; Aoki-Kinoshita 2013, 2015; Kletter et al. 2013; Agravat et al. 2014; Ichimiya et al. 2014).

This is the first version of the commentary on the MIRAGE guidelines for a glycan array experiment. Hopefully, the reviewers and editors of leading scientific journals will adopt the minimum information suggested by MIRAGE so that MIRAGE-supportive public repositories and databases can be established. Future versions will conform to progress in the technologies and analyses as well as wisdom from experience gained in the glycan microarray community. By analogy with other large-scale experiments in life sciences, data sharing and analysis tools will need to be developed and made available to researchers for comparing data across different laboratories. It is hoped that such an approach will become the norm for glycan arrays so that data presentation and publication standards are developed and lend themselves to annotation. We shall look forward to having comments and suggestions from the scientific research community, and will ensure that there will be effective routes for transmitting these for our attention.

## Availability

This manuscript describes the glycan microarray guidelines (Version 1.0) as of June 2016. The current versions of all MIRAGE guidelines and examples are available on the MIRAGE project website (http://www.beilstein-institut.de/en/projects/mirage/guidelines): sample preparations guidelines (doi:10.3762/mirage.1), MS guidelines (doi:10.3762/mirage.2) and glycan microarray guidelines (doi:10.3762/mirage.3).

## Supplementary Material

Supplementary Data
