# Supplementary material for: The minimum information required for a glycomics experiment (MIRAGE) project: improving the standards for reporting glycan microarray-based data
Source: Glycobiology. 2016 Dec 19;27(4):280–4. doi: 10.1093/glycob/cww118 (PMC5444268; doi:10.1093/glycob/cww118)
Supplement: Supplementary Data [file MIRAGEGlycanArrayGuidelinesVersion10_22-June-2016_09-11-2016_submission.pdf]

MIRAGE Glycan Microarray Guidelines

Guidelines for reporting glycan microarray analysis data

Version 1.0, 22 June 2016

doi:10.3762/mirage.3

These guidelines were drafted to be intentionally minimal and apply only to information required for generating interpretable data from a glycan array experiment. We have identified 8 areas, numbered in the workflow shown below, that are required for generating an unambiguous glycan array in the first instance and obtaining binding data. For each numbered component of the workflow area we provide guidelines for the minimal information that should be provided in reporting results.

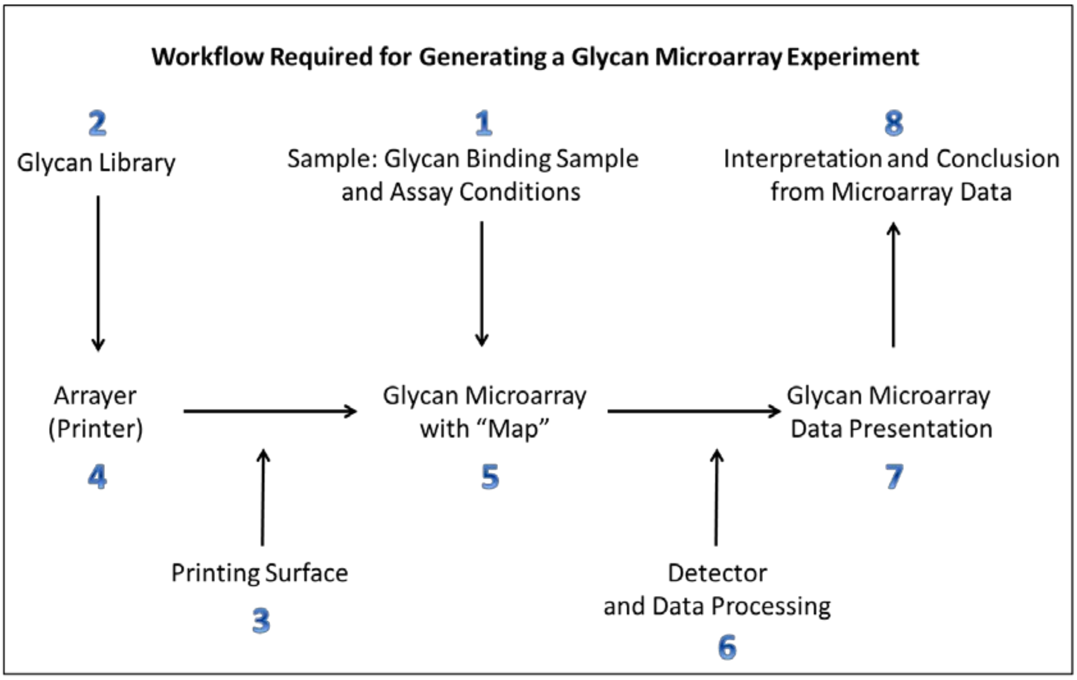

Similar to DNA microarray experiment, there are three levels of data relevant to a glycan microarray experiment:

Level 1: Raw data (original TIFF image from array scanner)

Level 2: Quantified numerical data output file (e.g. proscan or gpr) from scanner software

Level 3: Processed data (after association with glycan identities, averaging of replicates with appropriate statistics, data selection procedures, filtering and sorting, etc.)

The present MIRAGE guidelines suggest that only 'Level 3' data need be presented in published papers but also suggest that 'Level 1' and 'Level 2' data be retained by researchers so that it be available when suitable databases and processing software become accessible and that deeper data comparison and mining can be undertaken where worthwhile.

Classification	Guidelines
**1. Sample: Glycan Binding Sample**	
A wide variety of samples can be applied to glycan microarrays. Here we use the term 'Glycan Binding Sample' ['Sample'] for a protein, a microorganism or other molecule being analysed for carbohydrate recognition. Samples may include but are not restricted to glycan binding proteins (such as lectins, antibodies, adhesion molecules, and carbohydrate-binding modules), glycan binding organisms (such as viruses, fungi, bacteria, and animal cells), and other binding systems that may have affinity for glycans including aptamers, or other synthetic glycan binding molecules.	
Description of Sample	The Sample description should include an appropriate name, previous references, public database IDs (if available), its origin (synthetic, natural, or recombinant), and its source including vendor or method of preparation.<br>For organisms, the protocol for propagation should be included.<br>For proteins, a statement of purity is required and may represent a quantitative or qualitative description; i.e., % purity based on a specific assay (quantitative) or a component of a mixture such as a polyclonal antibody in a serum sample (qualitative). Fusion constructs for recombinant proteins for the purpose of detecting should also be described.
Sample modifications	If sample is directly labelled, describe the method and reagents (including sources) used for chemical labelling of the Sample, e.g. fluorescein, biotin, etc.
Assay protocol	The protocol used for microarray binding analysis of Sample should include Sample concentration. Describe the composition of solutions, time and temperature used for blocking (preventing non-specific binding) binding, and washing, as well as application of secondary reagent(s) required for the analyses. If the Sample is precomplexed with secondary detection reagents prior to adding to the array (e.g. anti-His antibody with his-tagged Sample) give the ratio of reactants and pre-complexing time and temperature.
**2. Glycan Library**	
Glycan arrays may be comprised of monosaccharides, oligosaccharides, polysaccharides or other glycoconjugates (glycolipids or glycoproteins). Glycans and glycoconjugates can be structurally defined; alternatively they can be purified or partially purified where structures are unknown ("shotgun" glycan arrays), or partially or on their way to being defined ("Designer" or "boutique" glycan arrays). Regardless of type, the glycan array is generated from a glycan library. Glycans are placed in designated areas of a "source plate" in an appropriate printing buffer, the robotics of the arrayer or microarray printer transfers the glycan preparations to the printing surface. The glycans are the fundamental components of the glycan array and minimal information regarding the glycan library should include:	
Glycan description for defined glycans	For mono- and oligosaccharides, the structure of each glycan, i.e. the sequence, linkage and anomeric configuration of each monosaccharide should be indicated using an appropriate nomenclature, which may be textual or symbolic as in **SNFG** (doi: 10.1093/glycob/cwv091).

	When possible, accession numbers assigned by an authoritative informatics resource, such as the GlyTouCan glycan structure registry (https://glytoucan.org/) should be included. This can be presented in the table of microarray binding results (Part 7).<br><br>The requirements for identifying and documenting glycan structure are beyond the scope of these guidelines. The purity of each glycan should be indicated [**MIRAGE Sample Preparations Guidelines** (doi:10.3762/mirage.1) are recommended]. Sources/references of glycans should be specified. For polysaccharides, the structures of predominant repeating units should be provided, as appropriate.
Glycan description for undefined glycans	The source and process for generating glycans or glycoconjugates should be described. The purification scheme should be described (if purified by LC methods, please refer to **MIRAGE LC Guidelines** as they become available). Some indication of purity should be included; i.e., HPLC or MS profile of each printed glycan target or glycan fraction should be provided [for MS data, **MIRAGE MS Guidelines** (doi:10.3762/mirage.2) are recommended].<br><br>For polysaccharides, information of genus, species and subtype or serotype of the organism should be provided.
Glycan modifications	Methods/references for functionalization or derivatization of glycans, including the structures of linkers should be described. Any effects on the glycan structure; i.e., reductive amination of reducing monosaccharide, should be indicated. The purity of each glycan derivative should be indicated.
**3. Printing Surface; e.g., Microarray Slide**	
A wide variety of solid phases can be used to print glycan microarrays. As the utility of the data generated from a glycan array analysis will be related to the quality of the printed array, minimal information regarding a surface on which the array is printed should include:	
Description of surface	What type of surface is being used; i.e., microscope-glass slide, microtiter plate or other format.
Manufacturer	Manufacturers of coated microscope slides, microtiter plates or other types of surfaces will provide a general description of their product. Provide the name of the manufacturer or name provided by the vendor with appropriate description of the product.
Custom preparation of surface	If the slides were custom prepared/coated, indicate the initial source and description of the starting surface; i.e., glass material, microtiter plate, etc., and the protocol used to prepare the surface prior to sample deposition.
Immobilization of glycans may be accomplished by a variety of non-covalent and covalent mechanisms depending on the nature of the linker between glycan and surface of substrate.	
Non-covalent Immobilization	Describe the non-covalent immobilization method; e.g. adsorption of lipid-linked glycans to nitrocellulose, or neoglycoprotein conjugates to polystyrene, nitrocellulose, etc.

	Describe any specific handling of the glycan derivatives required prior to sample deposition, e.g. formulation by adding carrier reagents.
Covalent Immobilization	Describe the chemistry on the slide surface that enables the covalent immobilization of the glycan samples; e.g. NHS-ester or epoxide for amine functional groups, maleimide for sulfhydryl functional group.
**4. Arrayer (Printer)**	
Glycan microarrays are printed using a robotic liquid delivery system to deposit glycans on a printing surface. The utility of the data generated will be related to the quality of the printed array, thus minimal information regarding the arrayer or printer and the parameters of the printing process should include:	
Description of Arrayer	Describe the printing robot used to deliver the glycans onto the array surface - provide the name of the manufacturer and model of the instrument. If the instrument is not commercially available, provide sufficient information to indicate the instrument is comparable to a commercially available arrayer.
Dispensing mechanism	Delivery of sample to the printing surface is by contact or non-contact liquid delivery. Describe the delivery method used; include number and type of dispensing tips, nozzles, or pins, etc.
Glycan deposition	Indicate the approximate volume and number of replicates of each glycan that is deposited on the surface.
Printing conditions	Indicate the composition of the printing solution and the concentration of the glycan in the printing solution (single or more than one concentration). The physical conditions reported for array production should include temperature, humidity, reaction time for covalent coupling or adsorption, and post reaction treatment if applicable.
**5. Glycan Microarray with "Map"**	
Glycan microarrays can vary from a large single array to multiple subarrays on a single microscope slide, as well as microtiter plate formats or other geometries. In order to generate interpretable data, the physical location of the replicate deposits of glycans on the array must be identified and correlated to the average signal (commonly fluorescence intensity) generated by detection of Sample bound. Thus minimal information regarding the location of glycans on the array should include the following:	
Array layout	Specify the array geometry; e.g. single large array, subarrays, microtiter plate, etc. State the number of replicates of each glycan and the total number of printed spots in each array.<br><br>Please note that the 'detailed glycan map' is not required at present. Such data should be retained by researchers for potential future use when new central storage facilities and analysis software are available.
Glycan identification and	Identify glycans printed on the array; i.e., define the total number of glycans printed and provide a table with a name and structure (if

quality control	known) of every glycan on the array. This can be merged with the table of microarray binding results (Part 7).<br><br>As necessary, describe methods for validating the identities of glycans on the array; this could be in the form of binding data from the array using Samples with known specificities.
**6. Detector and Data Processing**	
The most common detection method currently used for glycan microarray analysis is microscope slide fluorescence scanning, and there are several commercial instruments available for this type of analysis. Glycan microarrays can be printed in a variety of other formats including microtiter plate, and possibly in other geometries in custom instruments. In addition, a variety of detection methods are possible including surface plasmon resonance (SPR) and MS readouts and more precise or more sensitive detection may be developed in the future. Independent of the technology, minimal information regarding the detection of Sample binding to glycans on the array should include the following:	
Scanning hardware	Describe the scanning hardware (manufacturer/model), including detection method; e.g., optical detection (fluorescence/colorimetric), surface plasmon resonance (SPR) imaging technique, etc.; MS detection [**MIRAGE MS Guidelines** (doi:10.3762/mirage.2) are recommended].
Scanner settings	The intensities of signals generated from Sample binding to individual glycans are a reflection of affinities or avidities of the interactions. Provide an indication of whether the scanner settings (scanning resolution, laser channel, PMT and scan power) are such that signals are in a linear range of the scanner's detector (no 'saturation of signals') and whether the scanning resolution is adequate for the sizes of sample spots.
Image analysis software	Describe the software used to analyse (quantify) the output scanner image, indicating the name, version and manufacturer used and any special features active in the software (i.e. data smoothing, normalization, etc.).
Data processing	Provide details of how data in the table of microarray binding results (Part 7) are generated and calculated, i.e., specific software, normalization method, data selection procedures, and parameters, statistical analysis (including how the data from glycan replicates on the array were handled in the statistical method), transformation algorithm and scaling parameters.<br><br>Please note that the raw scan images and quantitative output files from the scanner software (e.g. proscan or gpr files) are not required at present. Such data should be retained by researchers for potential future use when new central storage facilities and analysis software are available.

7. Glycan Microarray Data Presentation	
Results of glycan microarray analyses are generally presented as histograms that identify average signal intensities (binding of Sample) with corresponding glycans (Glycan Number), but also can be presented in other formats such as heat maps. Regardless of presentation method minimal information regarding display of data for the detection of Sample binding to glycans on the array should include the following:	
Data presentation	The table of microarray binding results should include the list of glycans present in the array (names and structures if known, machine-readable form, if possible), and binding intensities with an indication of precision; i.e., standard deviation or other appropriate statistical parameter, should be reported. Other visual displays may include histogram charts, heat maps, binding curves, bar graphs, etc.
**8. Interpretation and Conclusion from Microarray Data**	
A simple statement should be given on the glycan(s) bound. If multiple glycans are bound, the common features of the structures should be summarised.	
Data interpretation	If software or algorithms are used to interpret processed data, e.g. for motif analysis, information or references to the software used, software version, name of the algorithm, and derived data (if available) should be included.<br>If software or algorithms are not used, methods or criteria used for selecting binders/non-binders should be described.
Conclusions	Describe the glycan sequences bound.
